# Long-term treatment with intranasal insulin ameliorates cognitive impairment, tau hyperphosphorylation, and microglial activation in a streptozotocin-induced Alzheimer’s rat model

**DOI:** 10.1038/srep45971

**Published:** 2017-04-06

**Authors:** Zhangyu Guo, Yanxing Chen, Yan-Fang Mao, Tingting Zheng, Yasi Jiang, Yaping Yan, Xinzhen Yin, Baorong Zhang

**Affiliations:** 1 Department of Neurology, the Second Affiliated Hospital, College of Medicine, Zhejiang University, Hangzhou, Zhejiang, 310009, China

## Abstract

Recent evidence reveals that aberrant brain insulin signaling plays an important role in the pathology of Alzheimer’s disease (AD). Intranasal insulin administration has been reported to improve memory and attention in healthy participants and in AD patients. However, the underlying molecular mechanisms are poorly understood. Here, we treated intracerebroventricular streptozotocin-injected (ICV-STZ) rats, a commonly used animal model of sporadic AD, with daily intranasal delivery of insulin (2 U/day) for 6 consecutive weeks and then studied their cognitive function with the Morris water maze test and biochemical changes via Western blotting. We observed cognitive deficits, tau hyperphosphorylation, and neuroinflammation in the brains of ICV-STZ rats. Intranasal insulin treatment for 6 weeks significantly improved cognitive function, attenuated the level of tau hyperphosphorylation, ameliorated microglial activation, and enhanced neurogenesis in ICV-STZ rats. Additionally, our results indicate that intranasal delivery of insulin probably attenuates tau hyperphosphorylation through the down-regulation of ERK1/2 and CaMKII in the brains of ICV-STZ rats. Our findings demonstrate a beneficial effect of intranasal insulin and provide the mechanistic basis for treating AD patients with intranasal insulin.

Alzheimer’s disease (AD) is the most prevalent neurodegenerative disorder and is characterized by progressive memory loss and cognitive impairment. The pathological features of AD are the accumulation of extracellular senile plaques that consist of the amyloid beta peptide (Aβ) and intracellular neurofibrillary tangles (NFTs) composed of abnormally hyperphosphorylated tau protein, particularly in the cerebral cortex and hippocampus. A large proportion of AD cases are late onset and sporadic. Owing to its multifactorial pathogenesis, reliable treatment remains unavailable so far.

Accumulating evidence from epidemiological studies shows that Type 2 diabetes (T2D) may be one of the major risk factors associated with AD^1^,^2^. Brain atrophy, energy metabolic dysfunction, insulin resistance, inflammation, and mitochondrial dysfunction are common features in AD and T2D^3^. Cerebral glucose metabolic dysfunction and deregulated brain insulin signaling are thought to be early brain abnormalities in AD^4^. Insulin signaling is involved in numerous brain functions, including energy metabolism, neural plasticity and cognition^5^. Recent evidence reveals that aberrant brain insulin signaling contributes to the pathogenesis of AD^6^,^7^. Thus, enhancing insulin signaling in the brain may be a promising therapeutic for the treatment of AD. Intravenous insulin infusion via the hyperinsulinemic-euglycemic clamp method has been shown to improve memory performance and attention in humans^8^,^9^. The intranasal delivery of insulin has also been reported to improve memory in healthy participants and in patients with AD and mild cognitive impairment (MCI)^10^,^11^. However, the mechanisms underlying the improved memory in AD and MCI patients with insulin treatment remain unclear.

Rats that received an intracerebroventricular administration of streptozotocin (ICV-STZ), develop several Alzheimer-like pathological changes and behavioral abnormalities, and have been used as a sporadic Alzheimer’s rat model^12^. More widely known as a diabetogenic substance, STZ causes severe neuronal dysfunction when injected into the brain at a subdiabetogenic dose that does not affect the glucose level in the brain^12^. Single or double ICV-STZ administration chronically decreases cerebral glucose uptake and leads to several AD-like pathophysiological changes such as the progressive deterioration of memory function, brain insulin resistance, decreased brain glucose metabolism, synaptic dysfunction, the accumulation of Aβ, and the hyperphosphorylation of tau^12^,^13^. The presence of brain insulin resistance and the hyperphosphorylation of tau found in this model makes it very useful for investigating the effects of insulin on tau hyperphosphorylation.

Our previous work showed that intranasal delivery of insulin for 7 days increased synaptic protein expression and reduced Aβ levels and microglial activation in the brains of 3 × Tg-AD mice, but did not affect the phosphorylation levels of tau^14^. In another study, we found that intranasal insulin (7 days) attenuated propofol-induced hyperphosphorylation of tau in 3 × Tg-AD mice^15^. To further investigate the potential beneficial effect of intranasal insulin on AD, especially sAD, we treated ICV-STZ rats with daily intranasal insulin for 6 consecutive weeks and studied the behavioral performance of these rats as well as the biochemical changes in the brain. Our results showed that intranasal insulin could improve cognitive deficits in ICV-STZ rats, which may be associated with reduced tau hyperphosphorylation, ameliorated activation of microglia and increased neurogenesis.

## Results

### Intranasal insulin rescues ICV-STZ induced memory deficits in rats

**Figure 1:**
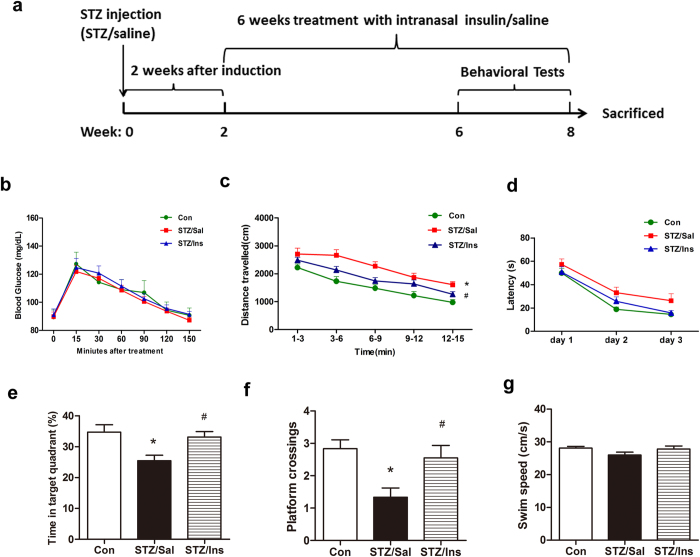
Effect of intranasal insulin on open field and Morris water maze performance. (**a**) Experiment schedule. (**b**) The level of blood glucose of the three groups during the treatment. (**c**) Spontaneous locomotor and exploratory activity was tested in an open field arena and the total distance travelled in the open field was recorded. (**d**–**g**) Spatial learning and memory were assessed with the Morris water maze. In 3-day acquisition trials, the rats’ escape latencies (**d**) and the average swim speed (**g**) were measured. In the probe trial, the time in the target quadrant in 60 s (**e**) and the number of platform site crossings (**f**) were recorded. Data are expressed as the means ± SEM (n = 13–15 per group). *P < 0.05 versus the control group; ^#^*P* < 0.05 versus the STZ/Sal group.

Intranasal delivery of insulin is reported to have no effects on peripheral glucose levels^10^,^16^. Similarly, we did not observe any changes in the level of blood glucose in animals treated with intranasal insulin (Fig. 1b). The apparent increase and subsequent decrease in blood glucose levels is a result of stress in response to handling. The general behavior of the rats was evaluated before the investigation of cognitive function. We checked the spontaneous locomotor and exploratory activities of the rats in the open field task. We found that the ICV-STZ rats travelled a longer distance than the control rats in the open field arena (Fig. 1c), indicating increased exploratory activities. In contrast, intranasal insulin markedly reduced the exploratory activities of the ICV-STZ rats (Fig. 1c).

Previous studies, including ours, have demonstrated impaired learning and memory in ICV-STZ animals^17^,^18^. To evaluate whether intranasal insulin ameliorates the cognitive deficits of the ICV-STZ rats, we investigated the spatial reference learning and memory of the rats with a Morris water maze test. We observed that all rats were able to learn the platform location during the 3-day acquisition phase as evidenced by the reduced latency to locate the hidden platform (Fig. 1d). No significant group differences in mean escape latency were found in the 3-day acquisition trials (Fig. 1d). These results indicated that the ICV-STZ rats, at this stage, were indistinguishable from the control rats in encoding and remembering the spatial coordinates of the platform within the environment. The retrieval of spatial memory was evaluated with a probe trial, which was carried out 24 hours after the last acquisition trial. We found that ICV-STZ rats spent less time in the former platform quadrant (the target quadrant) and showed fewer crossings over the former platform site (Fig. 1e,f), indicating impaired spatial memory in these rats. A six-week intranasal insulin treatment dramatically increased the time spent in the target quadrant and the platform site crossings of the ICV-STZ rats (Fig. 1e,f). These parameters of intranasal insulin treated ICV-STZ rats were comparable to those of the control rats (Fig. 1e,f). However, the differences observed above were not a consequence of the varying velocity of the rats, as the swim speed was not significantly different among groups (Fig. 1g). These findings indicated that intranasal treatment with insulin can successfully prevent spatial memory impairment in ICV-STZ rats.

### Intranasal insulin reduces tau phosphorylation in the brains of ICV-STZ rats

**Figure 2:**
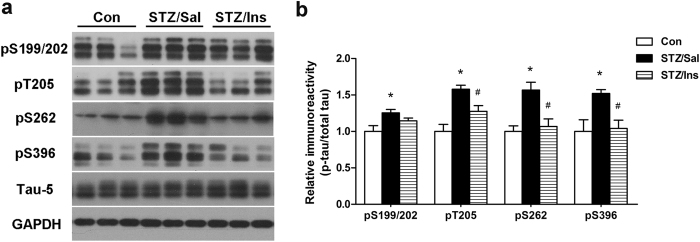
Effect of intranasal insulin on the phosphorylation of tau. (**a**) Hippocampal extracts were measured via Western blots. (**b**) Densitometric quantification of the blots after normalization to the total tau (Tau-5) level. Data are expressed as the means ± SEM (n = 9–11 per group). *P < 0.05 versus the control group; ^#^*P* < 0.05 versus the STZ/Sal group.

Abnormal hyperphosphorylation of tau is one of the most important pathophysiological features in AD and occurs in the brains of ICV-STZ rats^17^,^19^,^20^. Thus, we evaluated whether intranasal insulin treatment can ameliorate the accumulation of hyperphosphorylated tau in ICV-STZ rats via Western blots. As expected, we observed a marked increase in tau phosphorylation at all of the sites studied, including Ser199/202, Thr205, Ser262 and Ser396, but no change in the total tau level (Tau-5) in the hippocampus of ICV-STZ rats compared to that of control rats. Excitingly, intranasal delivery of insulin to the ICV-STZ rats greatly attenuated the hyperphosphorylation of tau at these sites with the exception of Ser199/202 (Fig. 2a,b). The level of tau phosphorylation in intranasal insulin treated ICV-STZ rats returned to a similar level to that of the control rats. These results indicate that long-term treatment with intranasal insulin can effectively reduce tau hyperphosphorylation in the brains of ICV-STZ rats.

### Intranasal insulin down-regulates tau kinases in the brains of ICV-STZ rats

**Figure 3:**
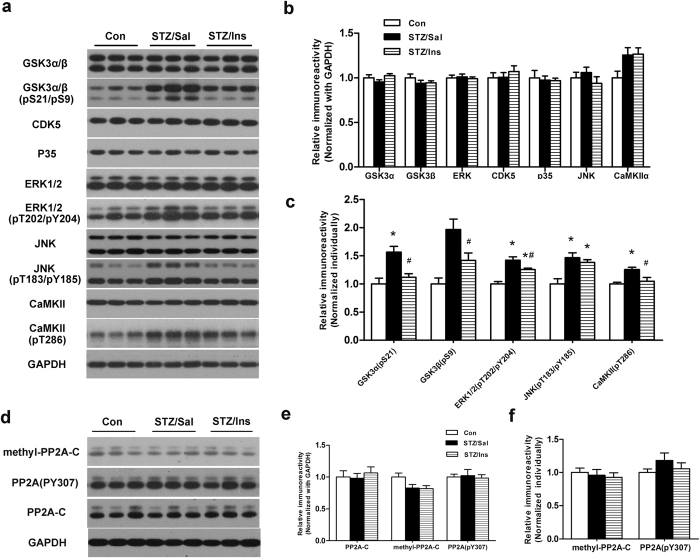
Effect of intranasal insulin on tau kinases and phosphatases. Hippocampal homogenates were detected via Western blots (**a**,**c**) and quantitative analysis was normalized to the GAPDH level (**b**,**e**) and the corresponding total protein kinase level (**c**,**f**). Data are expressed as the means ± SEM (n = 9–11 per group). *P < 0.05 versus the control group; ^#^*P* < 0.05 versus the STZ/Sal group.

It is well known that the phosphorylation of tau is related to the activities of its kinases and protein phosphatases^21^. Although the hyperphosphorylation of tau is well established in the brains of ICV-STZ animals, the levels and activities of these kinases and phosphatases are rarely known. To study the mechanism underlying the increase in tau phosphorylation, we next examined the levels of total and activated forms of specific tau kinases as well as the major tau phosphatases involved in the regulation of tau phosphorylation. Among all the tau kinases, glycogen synthase kinase-3 (GSK-3), cyclin-dependent kinase 5 (cdk5) and its activator p35, mitogen-activated protein kinase/extracellular signal-regulated kinase (MAPK/ERK), c-Jun N-terminal kinase (JNK), and calcium/calmodulin-dependent protein kinase II (CaMKII) are the major tau kinases implicated in AD^22^. The activation of these kinases is determined by their phosphorylation levels. With the exception of GSK-3, in which the phosphorylation of serine inhibits its activity, the other kinases are activated by their individual phosphorylation. We did not observe any significant differences in the total levels of these tau kinases among groups (Fig. 3a,b). Consistent with our previous studies^18^, the inhibitory phosphorylation of GSK-3 at Ser9 (GSK-3β) and Ser21 (GSK-3α) was dramatically upregulated in the hippocampus of ICV-STZ rats. Intranasal insulin reduced the phosphorylation level of GSK-3 in the hippocampus of ICV-STZ rats to that of the control rats (Fig. 3a,c). It was apparent that the activation status of GSK-3 was not responsible for the upregulation/downregulation of tau in vehicle/insulin treated ICV-STZ rats. We also evaluated the activation of other kinases (ERK1/2, JNK and CaMKII) in the hippocampus of the ICV-STZ rats. We found that ERK1/2, JNK and CaMKII were activated in ICV-STZ rats as evidenced by the marked increase in the phosphorylation levels of these kinases (Fig. 3a,c). Daily intranasal insulin treatments for 6 weeks decreased the phosphorylation levels of ERK1/2 and CaMKII in the hippocampus of ICV-STZ rats, but the activation of JNK remained unchanged (Fig. 3a,c). Taken together, these findings indicate that the intranasal delivery of insulin probably reduces tau hyperphosphorylation via a down-regulation of ERK1/2 and CaMKII in the brains of ICV-STZ rats.

In addition to tau kinases, protein phosphatases also regulate tau phosphorylation. PP2A is the major tau phosphatase in the human brain and its activity is significantly decreased in the AD brain^23^. To investigate whether PP2A is also involved in insulin’s inhibitory effect on tau hyperphosphorylation in the brains of ICV-STZ rats, we investigated the level of the catalytic subunit of PP2A (PP2A-C) and its post-translational modifications. The methylation of PP2A-C at Leu309 (methyl-PP2A-C) enhances, while tyrosine-phosphorylation at Tyr307 (PP2A-C (pY307)) inhibits, the activity of the enzyme^24^,^25^. We found that neither the level or the activity of PP2A-C were decreased in the hippocampus of ICV-STZ rats compared to those in control rats, as evidenced by the similar levels of PP2A-C, methyl-PP2A-C and PP2A-C (pY307) (Fig. 3d–f). The intranasal administration of insulin also had no effects on PP2A-C (Fig. 3d–f). These results suggest that PP2A might not be involved in the hyperphosphorylation of tau induced by STZ, and the attenuation of tau phosphorylation in intranasal insulin treated ICV-STZ rats might not be a result of the increased activity of PP2A.

### Intranasal insulin attenuates microglial activation in the brains of ICV-STZ rats

**Figure 4:**
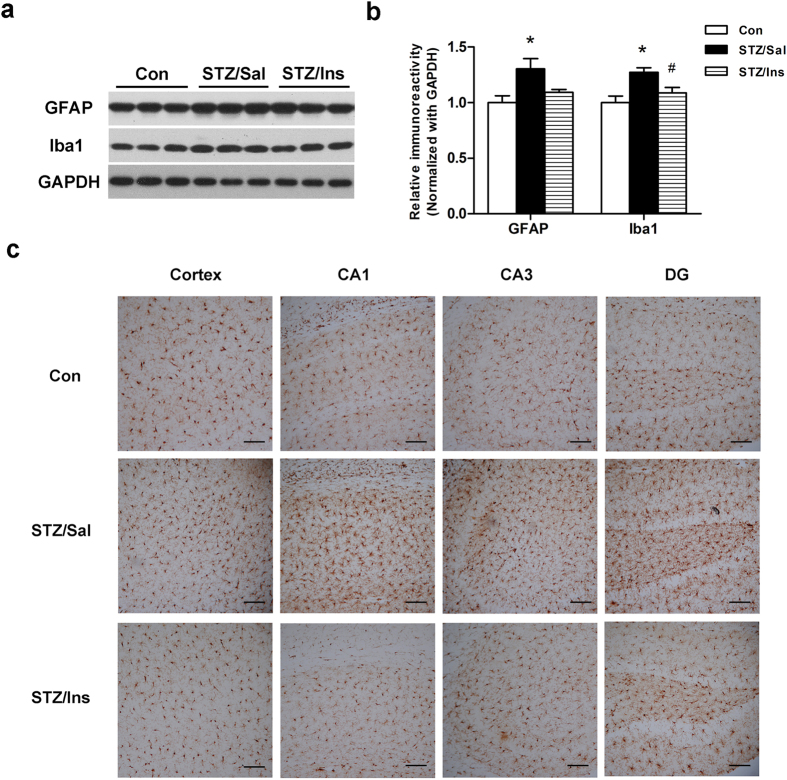
Effect of intranasal insulin on neuroinflammation markers. (**a**) The GFAP and Iba1 levels in whole hippocampus extracts were measured via Western blots. (**b**) Densitometric quantification of the blots after normalization to the GAPDH level. Data are expressed as the means ± SEM (n = 9–11 per group). *P < 0.05 versus the control group; ^#^*P* < 0.05 versus the STZ/Sal group. (**c**) Representative immunohistochemical staining of brain sections with an antibody against Iba1, n = 4 per group. Scale bar = 50 μm.

Neuroinflammation, as reflected by astrogliosis and microglial activation, is a pathological hallmark of AD that precedes plaque and tangle formation during AD progression^26^,^27^. The activation of astrocytes and microglia is also observed in ICV-STZ treated animals^17^,^28^. To investigate the effects of intranasal insulin on neuroinflammation in the hippocampus of ICV-STZ rats, we measured the level of glial fibrillary acidic protein (GFAP), the commonly used marker for astrocytes, and ionized Ca^2+^-binding adaptor molecule-1 (Iba1), a marker of microglia, via Western blots. Consistent with our previous findings, we observed a substantial increase in the level of GFAP and Iba1 in the hippocampus of ICV-STZ rats compared to control rats (Fig. 4a,b). Furthermore, the level of Ibal was markedly suppressed in intranasal insulin treated ICV-STZ rats than in vehicle treated rats. Immunohistochemical staining for Iba1 confirmed the over-activation of microglia in the hippocampus of the ICV-STZ rats, which was also ameliorated by treatment with intranasal insulin (Fig. 4c).

### Intranasal insulin regulates neurogenesis and the levels of synaptic proteins in the brains of ICV-STZ rats

**Figure 5:**
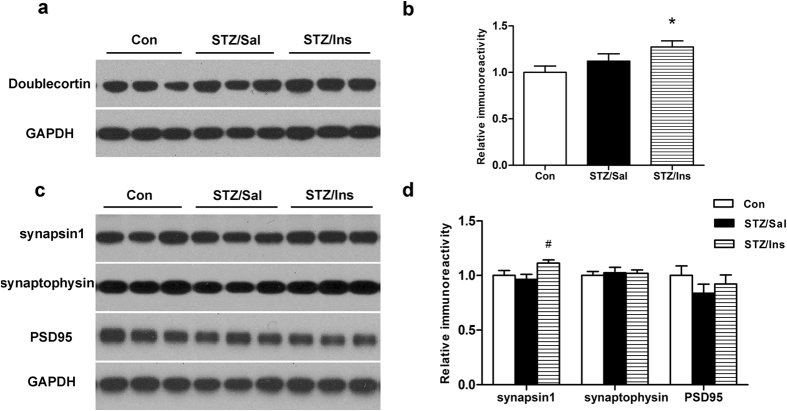
Effect of intranasal insulin on the levels of doublecortin and synaptic proteins. Hippocampus extracts were measured via Western blots (**a**,**c**) and quantitative analysis was normalized to the GAPDH level (**b**,**d**). Data are expressed as the means ± SEM (n = 9–11 per group). *P < 0.05 versus the control group; ^#^*P* < 0.05 versus the STZ/Sal group.

To evaluate the effects of insulin on neurogenesis, the levels of doublecortin (DCX), a marker of newborn neurons, were evaluated in the hippocampal homogenates of these rats. We did not observe a significant difference in the level of DCX in the hippocampus of ICV-STZ rats compared to control rats. However, intranasal insulin treatment dramatically upregulated the level of DCX in ICV-STZ rats compared to control rats (Fig. 5a,b), indicating that intranasal insulin might promote neurogenesis.

Synaptic dysfunction, which is already manifested at the early stage of the disease, has been demonstrated to correlate better with the severity of cognitive decline^29^. To investigate the possible underlying mechanisms by which insulin prevents the cognitive impairment of ICV-STZ rats, we evaluated the levels of pre- and post-synaptic proteins among these three groups. Although no significant differences were observed in the levels of synapsin 1, synaptophysin and PSD-95 (pre-, pre- and post-synaptic markers, respectively) in the hippocampus of ICV-STZ rats, we found that intranasal insulin treatment induced a modest but significant increase in the level of synapsin 1 in ICV-STZ rats (Fig. 5c,d). These findings suggest that synaptic proteins remain unchanged in these ICV-STZ rats and that intranasal insulin has very limited effects on enhancing the synaptic function.

### The effect of intranasal insulin on brain insulin signaling

**Figure 6:**
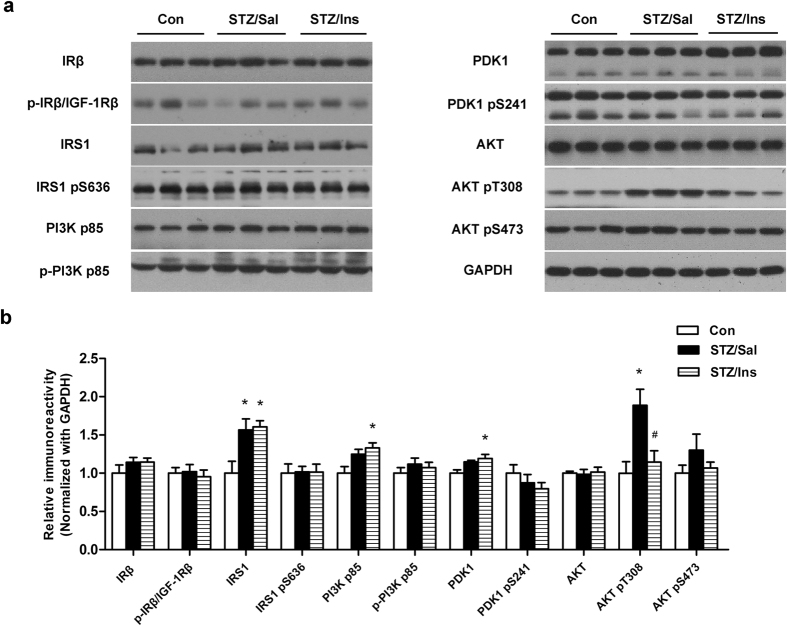
Effect of intranasal insulin on the brain insulin signaling pathway. (**a**) Hippocampal homogenates were detected via Western blots. (**b**) Quantitative analysis was normalized to the GAPDH level. Data are expressed as the means ± SEM (n = 9–11 per group). *P < 0.05 versus the control group; ^#^*P* < 0.05 versus the STZ/Sal group.

To explore the effects of intranasal insulin on brain insulin signaling, we assessed the level and activation of the key components involved, including insulin receptor β-subunit (IRβ), insulin-receptor substrate-1 (IRS1), phosphatidylinositide 3-kinases (PI3K), 3-phosphoinositide-dependent protein kinase-1 (PDK1), and protein kinase B (AKT). The activation of these proteins was assessed by measuring phosphorylation levels at activity-dependent sites. We found that the changes in the total and phosphorylated levels of most proteins were very mild in the hippocampus of ICV-STZ rats compared to control rats (Fig. 6a,b). Consistent with our previous studies^17^,^18^, the levels of phosphorylated AKT (AKT pT308) were substantially upregulated in ICV-STZ rats (Fig. 6a,b). The levels of IRS1, PI3K p85, and PDK1 in intranasal insulin treated ICV-STZ rats were also markedly stimulated compared to control rats, though these changes were insignificant compared to vehicle treated ICV-STZ rats (Fig. 6a,b). Intranasal insulin treatment restored the levels of AKT pT308 in the hippocampus of ICV-STZ rats (Fig. 6a,b). These results suggest that intranasal insulin treatment modestly boosts brain insulin signaling in the brains of ICV-STZ rats.

## Discussion

It is now widely recognized that brain insulin signaling plays a pivotal role in the central nervous system (CNS)^5^. Thus, targeting the brain insulin signaling pathway has become a promising therapeutic way to treat AD^30^. Although some small clinical studies have shown exciting outcomes of intranasal insulin on cognition improvement in MCI, AD and healthy participants, the underlying mechanisms remain elusive. Here, we investigated the effect of intranasal insulin on cognition and the pathological changes in the brains of ICV-STZ rats, a sporadic AD animal model. Although this model does not have the classic AD pathologies, amyloid plaques and neurofibrillary tangles, observed in models based on genetic mutations, it resembles many aspects of sporadic AD^13^. The etiology of sporadic AD is multi-factorial. Decreased brain glucose metabolism and brain insulin resistance are believed to be key contributing factors for sporadic AD^4^,^31^,^32^. Therefore, brain insulin resistance and subsequent hyperphosphorylation of tau, which are found in this model, are appropriate for the investigation of the beneficial role of intranasal insulin on tau hyperphosphorylation. We found that 6 weeks of intranasal delivery of insulin improved spatial memory in ICV-STZ rats, ameliorated tau pathologies and microglial activation and enhanced neurogenesis in the hippocampus of these rats. These findings provide a mechanistic basis for the treatment of AD with intranasal insulin.

Intranasal delivery of insulin is a non-invasive technique that bypasses the blood-brain barrier and delivers insulin from the nasal cavity to the CNS via intraneuronal and extraneuronal pathways^11^,^32^,^33^. Being devoid of hypoglycaemia and other peripheral metabolic effects, which limited the use of the systemic administration of insulin in non-diabetic patients, makes it a preferable way to delivery insulin into the brain. Apart from clinical studies, intranasal insulin has also been shown to improve cognition in control, diabetic, and AD transgenic mice^16^,^33^. In a recent study, we also found that pretreatment with intranasal insulin effectively prevented anesthesia-induced cognitive impairment^34^. In the present study, we further demonstrated the beneficial effects of intranasal insulin on cognition in sporadic AD rats. Six weeks of treatment with intranasal insulin improved spatial memory deficits in ICV-STZ rats as measured by Morris water maze tests. These results again confirmed the therapeutic potential of intranasal insulin on cognition.

One of the major pathobiochemical hallmarks in the brains of ICV-STZ animals is the hyperphosphorylation of tau^17^,^19^. Although one longitudinal study has shown the development of cerebral amyloid angiopathy in ICV-STZ rats^35^, little Aβ is detectable within 3 months after STZ injection^36^. Therefore, in this study, we only investigated the effect of intranasal insulin on tau hyperphosphorylation. Phosphorylation of tau at Ser199/202, Thr205, Ser262, and Ser396 was frequently observed in the brains of ICV-STZ animals in our previous studies and in studies from other groups^17^,^18^,^19^. The phosphorylation of these sites is also required for the conversion of normal tau to pathological tau^37^. In the present study, an obvious increase in all phosphorylation sites was observed in the hippocampus of ICV-STZ rats. In our recent studies, we found that pretreatment with intranasal insulin daily for 7 days attenuated anesthesia-induced hyperphosphorylation of tau in both wild type and 3 × Tg-AD mice^15^,^34^. However, we did not find any significant change in tau phosphorylation at any of the 10 sites studied in the brains of the 9-month-old 3 × Tg-AD mice with 7 days of intranasal insulin administration at the same dose. This discrepancy may be due to the relatively short period of intranasal insulin treatment, which is not enough to produce attenuation effects on already established tau hyperphosphorylation. The induction of the hyperphosphorylation of tau by anesthesia, on the other hand, can be mitigated by pretreatment with intranasal insulin for 7 days or even less. To investigate whether intranasal insulin can reduce tau hyperphosphorylation in AD animal models, we treated ICV-STZ rats with intranasal insulin daily for 6 weeks. We found that long-term delivery of intranasal insulin significantly reduced the hyperphosphorylation of tau, as observed in the hippocampus of ICV-STZ rats.

The phosphorylation state of tau is associated with the activities of its protein kinases and phosphatases, which are associated with the formation of tangles in AD brains^21^. In the present study, intranasal insulin had no effects on the levels of PP2A and its activity related modifications, but restored the dysregulation of tau kinases in the hippocampus of ICV-STZ rats. Each tau kinase phosphorylates tau at specific sites^21^,^22^. The tau phosphorylation sites measured in this study are involved in the conversion of normal tau into pathological tau^38^. To our surprise, the inhibitory phosphorylation of GSK-3β, one of the most relevant tau kinases in the AD brain, was upregulated in ICV-STZ rats. Our findings were consistent with previous studies in rodents and nonhuman primates that used an intraperitoneal delivery of STZ^37^,^39^, which suggested that GSK-3β is not involved in STZ-induced hyperphosphorylation of tau. Intranasal insulin, on the other hand, did not further upregulate, but downregulated the inhibitory phosphorylation of GSK-3β. These results suggest that GSK-3β might not be the responsible downstream factor of intranasal insulin’s effect on the attenuation of tau phosphorylation in ICV-STZ rats. However, the other two major tau kinases, ERK1/2 and CaMKII, which were significantly upregulated in ICV-STZ rats, were downregulated with intranasal insulin treatment. Ser199/202, Thr205, Ser262, and Ser396 on tau are phosphorylated by ERK1/2 or CaMKII^38^. The changed activity of these two kinases is consistent with the altered levels of tau phosphorylation at specific sites that we observed. These results suggest that intranasal insulin reduces tau hyperphosphorylation through the inhibition of tau kinases in ICV-STZ rats.

Neuroinflammation, as reflected by astrogliosis and microglial activation, is a double-edged sword in AD. At the early stage of AD, the activation of glial cells not only promotes the clearance and degradation of Aβ, but also regulates synaptic remodelling^40^,^41^,^42^,^43^. However, chronic activation of these glial cells triggers a persistent release of inflammatory mediators, which contribute to the progression of AD^44^. Activated microglia and astrocytes, surrounding or within the Aβ plaques, are evident in post-mortem brains of AD patients and animal models^40^,^45^. In agreement with previous reports^17^,^46^,^47^, we observed the activation of both microglia and astrocytes in the brains of ICV-STZ rats. Intranasal insulin effectively attenuated microglial activation, which was consistent with the results observed in insulin treated 3 × Tg-AD mice^14^. However, the mechanisms by which intranasal insulin exerts its anti-inflammatory role in the brain needs further investigation.

Neurogenesis plays a vital role in structural neuronal plasticity and network maintenance in the adult brain^48^, and altered neurogenesis in the adult hippocampus may exacerbate neuronal vulnerability and contribute to cognitive impairments in the course of AD^49^. Therefore, we compared the expression level of DCX, a marker of newborn neurons, among intranasal insulin- and saline-treated ICV-STZ rats and control rats. We found that intranasal insulin elevated the level of DCX in the brain of ICV-STZ rats compared to control rats, which suggested an increase in hippocampal neurogenesis. These results are consistent with our previous reports showing that intranasal insulin can enhance neurogenesis in the hippocampus of APP/PS1 mice^50^. Synapse loss is an early and invariant feature of AD and correlates well with cognitive decline^45^,^51^. The levels of pre- and post-synaptic proteins could reflect synapse function and neuroplasticity. In the present study, we did not find any significant changes in synaptic proteins after ICV-STZ treatment. The discrepancy between the expression of synaptic proteins and cognitive function has been reported before in aged rats^52^. The impaired memory of the ICV-STZ rats might be attributed to the loss of functional synapses or subtle changes in both the magnitude and distribution of effective synaptic coupling without causing substantial synapse loss^17^,^53^. Insulin receptors distribute at synapses and are extremely abundant in areas of rich synaptic density such as the cortex and hippocampus, suggesting a possible relationship between insulin signaling and synaptic plasticity^12^. In the present study, we found a modest increase in synapsin1, not synaptophysin or PSD-95, in the intranasal insulin treated ICV-STZ rats. These findings indicated that intranasal insulin might play a weak role in synaptic plasticity. The beneficial effects of insulin on cognition in these ICV-STZ rats may be through other mechanisms than by enhancing synaptic function.

It was reported that insulin signaling dysfunction correlated with an increased accumulation of Aβ, phosphorylated tau, reactive oxygen/nitrogen species, neuroinflammation and cognitive impairment in the brains of AD patients and animal models^54^,^55^. Several studies have demonstrated a deregulation in the expression of genes and/or proteins in the insulin signaling pathway^56^. We also assessed the total and phosphorylated levels of the key components of insulin signaling in the rat brain. Surprisingly, we did not observe marked changes in these ICV-STZ rats that were found previously in ICV-STZ treated mice^17^,^18^. This might be because in the present study, the rats were kept for a longer time after treatment with ICV-STZ. The levels of these components reached a balance via compensatory responses^17^,^57^. Furthermore, although the basal states of insulin signaling molecules were mildly changed, the response of the insulin signaling pathway to insulin might have already been compromised^31^. It would be very informative in the future to unveil the role of some molecules, especially IRS1 and AKT pT308, in this model.

In conclusion, we reported here that daily intranasal delivery of insulin for 6 weeks can ameliorate spatial memory impairments, reduce tau hyperphosphorylation and the activation of tau kinases, attenuate microglial activation, and promote neurogenesis in the ICV-STZ rat brains. As clinical trials found that intranasal insulin could also improve memory in healthy individuals^9^,^10^, it would be more informative and precise to include a control group treated with intranasal insulin, especially with regard to the role of intranasal insulin on synaptic proteins, neurogenesis, and the insulin signaling pathway. We will further investigate this aspect in the future to provide a more comprehensive understanding of the action of intranasal insulin in the brain. Nevertheless, our findings suggest that the neuroprotective effects of intranasal insulin on cognition observed in both animals and humans could be partially due to the alleviation of tau hyperphosphorylation, the attenuation of microglial activation, and the enhancement of neurogenesis. These results also provide experimental and mechanistic evidence that supports the progression of the use of intranasal insulin for AD patients to clinical trials.

## Methods

### Antibodies and reagents

The primary antibodies used in this study are listed in Table 1. Horseradish peroxide (HRP) conjugated anti-mouse and anti-rabbit secondary antibodies were obtained from Maibio (Shanghai, China). The enhanced chemiluminescence (ECL) kit was purchased from Pierce (Rockford, IL, USA). The ABC staining system was purchased from Zhongshan Jinqiao Biotechnology (Beijing, China). Insulin (Humulin R) was purchased from Eli Lily (Indianapolis, IN, USA). Other chemicals were from Sigma (St. Louis, MO, USA).

### Animals and treatment

Adult male Sprague–Dawley rats (250–280 g, 3 months of age) were obtained from SLAC laboratory animal company (Shanghai, China) and were kept in our laboratory animal facility for at least 1 week before use. Rats were housed in a quiet, temperature and humidity controlled room with 12:12 h light/dark cycle and *ad libitum* access to food and water.

The rats were first anesthetized and restrained onto a stereotaxic apparatus, then the following coordinates were used for ICV injection: −0.8 mm AP/+1.5 mm ML to Bregma and −3.6 mm DV to dura. STZ (3 mg/kg) dissolved in 10 μl sterile saline was injected slowly into the right lateral ventricle to produce the ICV-STZ rat model. The control rats were treated identically but with vehicle (saline). Two weeks after one single ICV injection of STZ, the rats received repeated intranasal insulin or saline daily for 6 consecutive weeks.

Intranasal delivery was performed manually without anesthesia while the rat head was hand-restrained in a supine position with the neck in extension, as previously described^16^,^58^. All rats were habituated to handling for 14 days prior to the experiment. A total of 2 U/50 μl insulin or saline were administered over alternating nares by a 10 μl Eppendorf pipette. The rat was held for an additional 5–10 s to ensure that the fluid was inhaled. Successful nasal delivery with this approach was confirmed by the presence of ink in the autopsied brains after nasal delivery with ink using the same approach. Four weeks later, rats were subjected to a battery of behavioral tests (Fig. 1a). Finally, the rats were decapitated and the brains were rapidly dissected. The hippocampus was dissected out, flash frozen separately in dry ice and stored at −80 °C for biochemical analysis. Some of the brains were fixed with buffered 4% paraformaldehyde. Sagittal sections (30 μm-thick) of the fixed brain tissue were prepared for immunohistochemistry. The sections were stored in glycol anti-freeze solution (ethylene glycol, glycerol, and 0.1 M PBS) at −20 °C until immunohistochemical staining.

In this study, we included 13 control rats, 14 intranasal saline treated ICV-STZ rats (STZ/Sal), and 15 intranasal insulin treated ICV-STZ rats (STZ/Ins) for all behavioral tests, 9–11 rats per group for biochemical analysis and 4 rats per group for immunohistochemical analysis.

### Open field test

Four weeks after insulin or vehicle treatment, spontaneous and exploratory activities of the animals were evaluated in an open field test. Each rat was placed in the center of a square cage (1 m × 1 m, with walls 40 cm high). The rats were allowed to freely explore the arena for 15 min. The locomotive activities of the rats (distance travelled) were recorded by a video camera. The arena was cleaned with 70% ethyl alcohol between tests. Each rat was tested only once.

### Morris water maze test

The Morris water maze (MWM) task was performed 5 weeks after the insulin or vehicle treatment. The maze consisted of a circular pool (150 cm in diameter, 60 cm in height) filled with water (temperature at 22 ± 1 °C). The pool was conceptually divided into four equal quadrants by imaging lines. A black platform (diameter, 10 cm) was positioned in the middle of the target quadrant, half-way between the center and the wall, and submerged approximately 1.0 cm below the surface of the water. For spatial acquisition, all rats were trained to find the platform and underwent four trials per day for 3 consecutive days. All animals entered the water facing the wall of the pool. The starting quadrant was changed each day. If the rat failed to find the hidden platform within 90 s, it was guided to the platform by the experimenter. The rats were allowed to remain on the platform for 15 s. The probe trial was performed 24 h after the last acquisition trial. In this phase, the hidden platform was removed and rats were given 60 s to swim in the pool. The time to reach the platform (escape latency), the time spent in the target quadrant, the number of platform crossings, and swimming speeds were recorded with a computerized tracking system.

### Western blot analysis

Hippocampi were homogenized in ice-cold buffer containing 50 mM Tris–HCl (pH 7.4), 50 mM GlcNAc, 20 μM UDP, 2.0 mM EGTA, 2 mM Na_3_VO_4_, 50 mM NaF, 20 mM β-glycerophosphate, 0.5 mM AEBSF, 10 μg/ml aprotinin, 10 μg/ml leupeptin, and 4 μg/ml pepstatin A. Protein concentrations of the homogenates were measured with the bicinchoninic acid (BCA) protein assay kit (Pierce, Rockford, IL, USA). Equal amounts of protein were separated by SDS-polyacrylamide gel electrophoresis and electro-transferred to PVDF membranes (Millipore, Bedford, MA, USA), which were subsequently blocked by 5% non-fat milk in 0.1% Tris-buffered saline/Tween-20 (TBST) for 1 h. The membranes were incubated with appropriate primary antibodies overnight at 4 °C and then incubated with the appropriate horseradish peroxidase-conjugated secondary antibody (1:5000) for 1 h at room temperature. Immunoreactive bands were visualized with enhanced chemiluminescent (ECL, Pierce, and Rockford, IL) and analysed with the Multi Gauge V3.0 software (Fuji Photo Film Co., Ltd, Edison, NJ, USA).

### Immunohistochemical staining

Immunohistochemical staining was performed according to our previously described methods^14^,^15^. Briefly, free floating sections were incubated for 20 min with 0.3% H_2_O_2_ and 0.3% Triton X-100 for 15 min at room temperature, washed in PBS, and blocked in a solution containing 5% normal goat serum and 0.1% Triton X-100 for 30 min. Sections were then incubated overnight with primary antibody at 4 °C. Normal goat serum in the absence of primary antibody was used as a negative control. Sections were washed and incubated with horseradish peroxidase-conjugated secondary antibody and visualized with a stable diaminobenzidine/hydrogen peroxide solution. The stained sections were mounted on microscope slides, dehydrated through graded alcohols, and coverslipped with neutral balsam.

### Statistical analysis

Data are presented as the means ± SEM, and *P* < 0.05 was considered statistically significant. For distance travelled in the open field and escape latency during the MWM, a two-way repeated-measures analysis of variance (ANOVA) was used with Fisher’s LSD *post hoc* tests. For other data, a one-way ANOVA followed by Bonferroni *post hoc* test was utilized for comparisons among various groups. All statistical analyses were performed using Prism (GraphPad Software Inc., San Diego, CA, USA).

### Ethics statement

All animal experiments were carried out in accordance with the National Institutes of Health guide for the care and use of laboratory animals (NIH Publication No. 85-23, revised 1996), and protocols were approved by the Institutional Animal Care and Use Committee of Zhejiang University.

## Additional Information

**How to cite this article:** Guo, Z. *et al*. Long-term treatment with intranasal insulin ameliorates cognitive impairment, tau hyperphosphorylation, and microglial activation in a streptozotocin-induced Alzheimer's rat model. *Sci. Rep.* **7**, 45971; doi: 10.1038/srep45971 (2017).

**Publisher's note:** Springer Nature remains neutral with regard to jurisdictional claims in published maps and institutional affiliations.

## Figures and Tables

**Table 1:** Primary antibodies used in this study.

Antibody	Phosphorylation sites	Source
pS199/202	Ser199/202	Invitrogen, Grand Island, NY
pT205	Thr205	Invitrogen
pS262	Ser262	Invitrogen
pS396	Ser396	Invitrogen
Tau-5		Millipore, Temecula, CA, USA
GSK-3α/β		Cell Signaling Technology, Danvers, MA
GSK-3α/β pS21/9	Ser21/9	Cell Signaling Technology
CDK5		Santa Cruz Biotechnology, Santa Cruz, CA
P35		Cell Signaling Technology
ERK1/2		Cell Signaling Technology
ERK1/2(pT202/pY204)	Thr202/Tyr204	Cell Signaling Technology
JNK		Cell Signaling Technology
JNK(pT183/pY185)	Thr183/Tyr185	Cell Signaling Technology
CaMKII		Santa Cruz Biotechnology
CaMKII (pT286)	Thr286	Santa Cruz Biotechnology
Methyl-PP2A-C		Millipore
PP2A(pY307)	Tyr307	Santa Cruz Biotechnology
PP2A-C		Millipore
GFAP		Millipore
Iba1		Wako Chemicals, Richmond, VA, USA
Doublecortin		Santa Cruz Biotechnology
Synapsin 1		Santa Cruz Biotechnology
Synaptophysin		Millipore
PSD95		Cell Signaling Technology
IRβ		Cell Signaling Technology
p-IRβ/IGF-1Rβ	Tyr1150/1151(IRβ), Tyr1135/1136 (IGF-1Rβ)	Cell Signaling Technology
		
IRS1		Cell Signaling Technology
IRS1 pS636	Ser636	Santa Cruz Biotechnology, Santa Cruz, CA
PI3K p85		Cell Signaling Technology
p-PI3K p85	Tyr458	Cell Signaling Technology
PDK1		Cell Signaling Technology
PDK1 pS241	Ser241	Cell Signaling Technology
AKT		Cell Signaling Technology
AKT pT308	Thr308	Cell Signaling Technology
AKT pS473	Ser473	Cell Signaling Technology
GAPDH		Santa Cruz Biotechnology
